# BDNF alleviates Parkinson's disease by promoting STAT3 phosphorylation and regulating neuronal autophagy

**DOI:** 10.1007/s00441-023-03806-1

**Published:** 2023-07-14

**Authors:** Xin Geng, Yanghong Zou, Jinghui Li, Shipeng Li, Renli Qi, Hualin Yu, Lianmei Zhong

**Affiliations:** The Second Department of Neurosurgery, The First Affiliated Hospital of Kunming Medical University, Yunnan Provincial Clinical Research Center for Neurological Disease, No. 295 Xichang Road, Kunming, Yunnan 650032 China

**Keywords:** BDNF, STAT3 phosphorylation, Autophagy, Parkinson

## Abstract

Parkinson's disease (PD) is a neurodegenerative disorder characterized by the gradual death of dopaminergic neurons. Brain-derived neurotrophic factor (BDNF) and its receptors are widely distributed throughout the central nervous system, which can promote the survival and growth of neurons and protect neurons. This study revealed that BDNF promotes STAT3 phosphorylation and regulates autophagy in neurons. The PD mouse model was established by 1-methyl-4-phenyl-1,2,3,6-tetrahydropyridine (MPTP). Moreover, SH-SY5Y cells were treated with 1-methyl-4-phenyl-pyridinium (MPP^+^) to establish a PD cell model. The level of BDNF was low in PD model mice and SH-SY5Y cells treated with MPP^+^. BDNF enhanced the levels of p-TrkB, P-STAT3, PINK1, and DJ-1. BDNF promoted autophagy, inhibited the level of p-α-syn (Ser129) and enhanced cell proliferation. The autophagy inhibitor 3-Methyladenine (3-methyladenine, 3-MA) reversed the protective effects of BDNF on neurons. BiFC assay results showed that there was a direct physical interaction between BDNF and STAT3, and coimmunoprecipitation experiments indicated an interaction between STAT3 and PI3K. The PI3K agonist Recilisib activated the PI3K/AKT/mTOR pathway, promoted autophagy, and alleviated neuronal cell damage. BDNF alleviates PD pathology by promoting STAT3 phosphorylation and regulating neuronal autophagy in SH-SY5Y cells and cultured primary neurons. Finally, BDNF has neuroprotective effects on PD model mice.

## Introduction

Parkinson's disease (PD) is a neurodegenerative disease characterized by the gradual loss of dopaminergic neurons in the substantia nigra (SN) and the accumulation of cytoplasmic a-synuclein (a-syn) into Lewy bodies and is the second most common neurodegenerative disease at present (Poewe et al. 2017). Its main clinical signs and symptoms are motor dysfunction and resting tremors (Cabreira and Massano 2019). Currently, the pathogenesis of PD is not clear, and the main view is that it is related to abnormal protein aggregation, mitochondrial dysfunction, and oxidative stress (Borsche et al. 2021). This study explores the potential therapeutic targets of PD at the molecular level.

Brain-derived neurotrophic factor (BDNF) is a member of the neurotrophic factor family and promotes neuroprotection and neuroregeneration. BDNF expression was confirmed in the hippocampus, frontal cortex, midbrain, amygdala, hypothalamus, striatum (ST), pons, and medulla oblongata (Palasz et al. 2020). Changed levels of BDNF in the circulation and central nervous system (CNS) have been shown to be associated with the pathogenesis of neurodegenerative diseases, including PD (Akbari et al. 2022; Palasz et al. 2020). There are data suggesting that decreased levels of BDNF may lead to the overexpression of α-syn and suppression of dopamine synthesis in PD patients (Fang et al. 2017; Kang et al. 2017; Katila et al. 2017). Studies have made clear that α-syn aggregation is closely related to the pathogenesis of PD (Mehra et al. 2019). Notably, mutating the gene encoding BDNF in mice incapacitates dopaminergic neurons, confirming the role of BDNF in protecting neurons from damage and degeneration (Baker et al. 2005; Baquet et al. 2005; Gerecke et al. 2010). Kang et al. (2022) showed that stimulation of deficient BDNF/TrkB signaling with the small molecular agonist CF3CN increased the number of TH-positive dopaminergic neurons and increased motor functions in MPTP-induced human SNCA transgenic PD model mice. Fan et al. (Fan et al. 2020) demonstrated that knockdown of brain-derived neurotrophic factor anti-sense (BDNF-AS) could elevate SH-SY5Y cell viability and inhibit apoptosis in MPTP-induced PD model by regulating miR-125b-5p.

BDNF has a high affinity for the primary ligand tropomyosin-related kinase B (TrkB), which is expressed in the frontal cortex, hippocampus, cerebellar cortex, pituitary gland, visual system, and hypothalamus. BDNF interacts with it through the immunoglobulin constant 2 (Ig-C2) domain (Jin 2020). A previous study indicated that deoxygedunin, as a small molecule TrkB agonist, improved the behavioral performance of PD model mice and reduced dopaminergic neuron loss in the SN, displaying prominent neuroprotective properties (Nie et al. 2015). In addition, Effects of vagus nerve stimulation are mediated in part by TrkB in a PD model by chronically administering ANA-12 (a TrkB-specific antagonist) (Farrand et al. 2019). BDNF-TrkB binding regulates at least three intracellular signaling pathways (Björkholm and Monteggia 2016), including phosphatidylinositol-3'OH-kinase (PI3K), which can mediate autophagy through the AKT-mTOR pathway.

Impaired protein homeostasis and the accumulation of damaged or abnormally modified proteins are common characteristics in many neurodegenerative disorders, including PD. As one of the major degradation pathways, autophagy plays a pivotal role in maintaining the effective turnover of proteins and damaged organelles in cells (Hou et al. 2020; Lizama and Chu 2021). Xiaojuan Han demonstrated that kaempferol promotes neuroinflammatory inhibition via the cooperation of ubiquitination and autophagy to treat neurodegenerative disorders (Han et al. 2019). Jiaxin Yuan found that activation of TRPV1 channels in microglia enhances their autophagy ability to clear α-synuclein, enhancing PD therapy. The enzyme tyrosine hydroxylase and p-α-syn (Ser1229) of treated PD model mice almost recovered to the normal levels of healthy mice (Yuan et al. 2022). A study indicated that there is reduced chaperone-mediated autophagy activity in the brains of PD patients, providing evidence for the role of autophagy in PD pathogenesis and Lewy body formation (Alvarez-Erviti et al. 2010).

Signal transducer and activator of transcription 3 (STAT3) is a known transcription factor that mediates extracellular signals by interacting with polypeptide receptors on the cell surface (You et al. 2015). A study showed that resveratrol could indirectly upregulate the PI3K/AKT/mTOR pathway by revitalizing JAK2/STAT3 (Hou et al. 2018). Activation of the PI3K/AKT/mTOR signaling pathway plays a key regulatory role in autophagy (Xu et al. 2020). In acute PD, abnormal upstream activation of the PI3K/AKT/mTOR pathway and inflammasome activation were found to be associated with impaired autophagy (Giacoppo et al. 2017). Therefore, exploring the function and mechanism of STAT3 in modulating autophagy through the PI3K/AKT/mTOR pathway may be a new direction in the development of treatments for neurodegenerative diseases.

Studies have shown that the binding of BDNF and TrkB can activate various intracellular PI3K pathways to regulate autophagy (Numakawa et al. 2010). However, the role of BDNF and the PI3K pathway in PD pathology has not yet been reported. In addition, activation of the PI3K pathway plays a pivotal regulatory role in autophagy, and this pathway can be mediated by STAT3. Therefore, we speculate that BDNF may activate the STAT3-mediated PI3K/AKT/mTOR pathway to facilitate autophagy and alleviate PD pathology.

## Materials and methods

### PD mouse model

C57BL/6 mice (5 weeks old, 15–17 g) were obtained from the Animal Experimental Center of Kunming Medical University. The mice were housed under the standard conditions of appropriate temperature (23 ± 2 ℃), humidity (50 ± 5%) and 12 h brightness/12 h darkness cycle. They were fed a standard diet and had free access to drinking water. The mice were subjected to a 12 h fasting without water before operation. All experimental animals were given humane care following the 3 R principle, and the protocol was implemented in accordance with guidelines of the China Animal Science Standardization Technical Committee. The mice were randomly divided into 3 groups (n = 5): the NC group, PD group, and PD + oe-BDNF group. The mice were intraperitoneally injected with 0.2% MPTP solution (20 mg/kg/d, normal saline) for 5 days to establish a PD mouse model as previously described (Geng et al. 2019), while mice in the NC group were intraperitoneally injected with an identical volume of normal saline. To assess the role of BDNF in PD induced by MPTP, two days before constructing the PD mouse model, 2 μL of oe-BDNF (20 nM, GenePharma, China) was injected into the midbrain of PD model mice to overexpress BDNF.

### Pole climbing experiment

A cork ball was fixed on a wooden club with a diameter of 1 cm and a length of 50 cm. The mice were placed on the cork ball with the ball facing up and the wooden pole perpendicular to the ground. The time for the mice to reach the ground was recorded. Each mouse was tested five times.

### Grid experiment

A laboratory-made metal grid with a length of 50 cm and a width of 30 cm was used, and each grid was 1 cm^2^. The mouse was placed head up in the center of the grid, perpendicular to the ground, with all four limbs firmly on the grid. The time for the mice to move any paw was recorded, which was called descent latency (DL). Each experiment was repeated five times for each mouse.

### Immunohistochemistry

Mouse brain tissues were quickly collected after euthanasia. Brain tissue was fixed in 4% formalin, imbedded in paraffin, cut into 4 μm sections, and placed onto poly-L-lysine-coated glass slides. Slides were deparaffinized, dehydrated, dipped in sodium citrate buffer (pH = 6.0), preconditioned for 10 min in a microwave oven, and washed for 10 min with PBS. After blocking for 10 min with 3% hydrogen peroxide at room temperature, the slides were incubated overnight with LC3, BDNF and TH primary antibodies (Abcam, UK) at 4 °C. The corresponding secondary antibody was added and incubated. After reactivity with DAB chromogen, the slides were stained with hematoxylin and covered with glycerol gel, and the staining was observed and photographed under an inverted microscope.

### Nissl staining

The sections were dewaxed, flushed with distilled water, dyed with thionine aqueous solution, incubated at 60 ℃ for 30–60 min, rapidly differentiated with ethanol differentiation solution, and finally rapidly dehydrated with absolute ethanol, cleared and sealed. The glass slides were observed, and images were obtained under an optical microscope (Olympus, Japan).

### Measurement of dopamine levels

The dopamine (DA) levels in the striatum were measured by high-performance liquid chromatography (HPLC), as previously described (Wang et al. 2020). Briefly, the striatum was lysed and homogenized in 0.1 mL of 0.4 M perchloric acid for 40 min on ice, followed by centrifugation (12,000 rpm, 20 min) at 4 °C. After that, 80 μL of the supernatant was mixed with 40 μL of liquid B (20 mm citramalic acid–potassium, 300 mm dipotassium phosphate, 2 mm EDTA-2Na). The supernatants were collected to measure the DA level.

### Cell culture and treatment

The neuronal cell line SH-SY5Y (Pricella, China) was cultured in MEM (Gibco, USA). Primary neuronal cells were obtained as previously described (Geng et al. 2019). The ventral mesencephalon was dissected from E12.5 C57BL/6 embryos in cold PBS, and DMEM containing 0.25% trypsin was added to obtain a single cell suspension after adding DMEM/F12 (Gibco. USA) supplemented with 10% FBS. The cells were seeded on 96‐well plates coated with poly-D-lysine and cultured in B27-supplemented neurobasal medium (Gibco, USA).

When the cell growth density was approximately 80%, oe-BDNF, si-STAT3, and si-NC (GenePharma, China) were transfected using a Lipofectamine 2000 kit (Solarbio, China) into neuronal cells. The cells were cultured at 37 °C in a 5% CO_2_ cell incubator. After 24 h, the transfection efficiency was detected. Cells were treated with 1 mmol/L MPP^+^ for 24 h to establish a PD cell model. The NC group was cultured in DMEM substrate without drugs, and the experimental groups were cultured in DMEM substrate containing 5 mmol/L 3-MA (3-Methyladenine, PI3K inhibitor), 2 mmol/L Stattic (STAT3 inhibitor) or 5 mmol/L Recilisib (PI3K/Akt/mTOR agonist).

### Cell viability and apoptosis assay

Cells were seeded in 96-well plates at 4 × 10^4^ cells/mL and then cultured for 0, 12, 24 and 48 h. 20 µL of CCK-8 solution (Beyotime, China) was added to the wells according to the manufacturer's instructions. The cell viability was calculated by using a microplate reader at 450 nm.

Annexin V binding fluorescein isothiocyanate/propidium iodide (FITC/PI) flow cytometry was used to analyze apoptosis. Specifically, cells were collected using cold PBS and hatched with 10 μL Annexin-V-FITC/PI (Solarbio, China) for 15 min in darkness. Flow cytometric analysis was executed using a flow cytometer (Beckman, USA) and FlowJo software.

### BDNF level measurement

BDNF levels in the midbrain of mice or cell culture supernatant were examined using enzyme-linked immunosorbent assay (ELISA) according to the manufacturer's instructions (Promega, USA).

### RT‒qPCR

TRIzol was added to lyse the cells and extract the total RNA. A NanoDrop1000 spectrophotometer and agarose gel electrophoresis were used to determine the concentration and quality of RNA. Reverse transcription was performed as follows: The reaction mixture was 500 ng total RNA, 0.5 µL Randome 6 primer, 2 µL 5 X Mix and 0.5 µL oligo dT Primer, ddH_2_O supplemented to 10 µL. The reverse transcription reaction procedure was 37 ℃ for 15 min, 85 ℃ for 5 s, and held at 4 ℃. The RT‒qPCR instrument was run in a Bio-Rad CFX96 instrument. The total reaction mixture was 25 µL, incorporating 10 ng of cDNA, 12.5 µL of L SYBR Premix Ex Taq II and 10 µmol/L of each of the forward and downstream primers, and ddH_2_O was used to bring the total volume to 25 µL. The reaction routine was: 95 ℃ 30 s; 95 ℃ 5 s, 60 ℃ 30 s 39 cycles; 95 ℃ 10 s, 65 ℃ 6 s, 95 ℃ 5 s. GAPDH was used as an internal reference, and the gene level of the detection results was calculated according to the 2^−∆∆Ct^ method (Table 1).

**Table 1:** Primer sequences

Target	Sequence (F: Forward primer, R: Reversed primer)
BDNF	F:5'-TCTGACGACGACATCACTG-3' R:5'-CCGAACATACGATTGGGT-3'
GAPDH	F:5'-TGGCAAAGTGGAGATTGTT-3' R:5'-CTTCTGGGTGGCAGTGAT-3'

#### Western blot

Cells or brain tissues in each group were collected, RIPA buffer and 1 mM PMSF protease suppressant were added, and the samples were lysed on ice for 5 min and centrifuged at 12,000 × g for 10 min at 4 °C. The concentration of total protein in the supernatant was determined using a BCA kit. Total protein was isolated by SDS‒PAGE on a 10% gel (80 V for 30 min, 120 V for 60 min). Protein specimens were transferred to PVDF membranes (Bio-Rad, USA) at 110 V and then blocked with 5% skim milk for 2 h at room temperature. After washing the membrane, the membrane was incubated with the corresponding secondary antibody (1:2000, Abcam, UK) at room temperature for 2 h. ECL was used for color rendering, and ImageJ was used to analyze band gray values. The antibodies were as follows: p-TrkB (ab229908, 1:1000), TH (ab137869, 1:5000), p-α-syn (ab51253, 1:2000), PINK1 (ab186303, 1:1000), DJ-1 (ab76008, 1:5000), LC3 (ab192890, 1:2000), Beclin1 (ab207612, 1:2000), p62 (ab240635, 1:1000), p-STAT3 (ab76315, 1:20000), STAT3 (ab68153, 1:1000), p-PI3K9 (ab278545, 1:1000), PI3K (ab140307, 1:2000), p-AKT (ab38449, 1:1000), AKT (ab131168, 1:1000), p-mTOR (ab109268, 1:2000), mTOR (ab134903, 1:1000), and GAPDH (ab9485, 1:2500).

#### Autophagic flow was detected by autophagic double-labeled adenovirus (mRFP-GFP-LC3)

mRFP-GFP-LC3 lentivirus was used to label and track autophagic flux. In accordance with the instructions, neural cells were transfected for 48 h with fluorescent mRFP-GFP-LC3 lentivirus and cultured for 24 h according to the experimental requirements. Confocal fluorescence microscopy was conducted and images were acquired. The yellow signal (merging of GFP and RFP signal) represents the early autophagosome, while the red signal (RFP signal only) represents the late autolysosome. Autophagic flux was assessed by the color change of GFP/mRFP.

#### Immunofluorescence

Cells were grown on glass covers overnight to prepare cell slides, which were then fixed with 4% paraformaldehyde, incubated with 0.2% Triton X-100 for 15 min, and then incubated with 5% bovine serum albumin at room temperature for 1 h. The slides were treated with p-α-syn (ab51253, 1:2000), STAT3 (ab68153, 1:1000) or BDNF (ab108319, 1:2000) and incubated at 37 °C for 4 h. The secondary fluorescent antibody was added to the sample mixture and incubated for 2 h in the dark. DAPI (Beyotime, China) was used to stain nuclei and the slides were observed using fluorescence microscopy.

#### Bimolecular Fluorescence Complementation (BiFC)

Human STAT-3 cDNA and BDNF cDNA were inserted into BiFC vectors pBiFC-VC155 and pBiFC-VN173 (Sangon Biotech, China). Recombinant plasmids were co-transfected into cells. Cells were fixed with 4% paraformaldehyde, incubated with DAPI for 5 min, and observed and photographed under a confocal microscope (Nikon Instruments Inc) after 24 h, with excitation and emission wavelengths of 488 and 500 nm, respectively.

#### Coimmunoprecipitation (CO-IP)

Cultured cells were homogenized, and a protease inhibitor mixture (Beyotime, China) was added to hypotonic lysis buffer and lysed on ice for 15 min. The cell lysate was centrifuged for 10 min at 4 ℃. The microspheres were resuspended in high salt buffer (hypotonic buffer incorporating 420 mM sodium chloride and 25% glycerol), spun at 4 °C for 30 min, and the A/G agarose beads and antibody were incubated for 60 min at 4 °C and then washed twice. Nonspecific IgG (Shanghai Jining Industrial Co., Ltd., China) was used as a control. The agarose beads were centrifuged to the lower part of the tube for 3 min at 3 000 rpm and 4 ℃ after immunoprecipitation. The supernatant was collected, and the agarose beads were washed 3–4 times with 1 mL of lysis buffer and resuspended in 30 mL of specimen buffer. The sample was heated for 2 min, and 20 mL of precipitated protein was added into each channel of the SDS‒PAGE gel. After washing, the corresponding HRP-labeled secondary antibody was added, incubated for 1 h at room temperature, and developed with ECL chemical solution.

#### Statistical analysis

GraphPad Prism 8.0 was used to analyze the experimental data and plot the graphs. The analysis results are expressed as the mean ± SD. One-way analysis of variance and t tests were used, and P < 0.05 was considered statistically significant.

## Results

### BDNF alleviates damage and is associated with autophagy in PD cell models

**Fig. 1:**
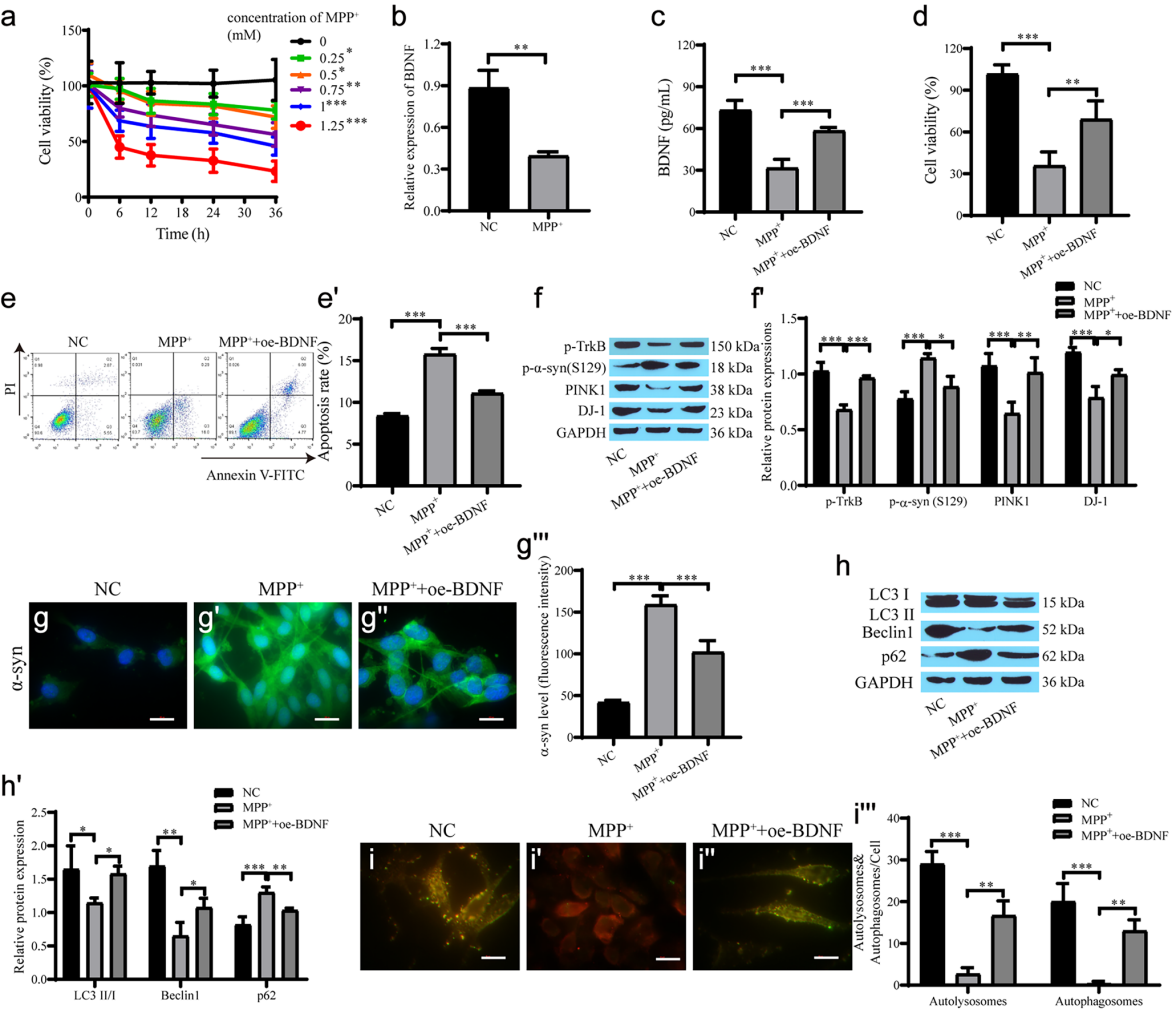
BDNF alleviates cell damage in the PD cell model and is related to autophagy. **a** CCK-8 for cell proliferation treated with different concentration of MPP^+^; **b** RT‒qPCR for BDNF expression; **c** ELISA for secreted BDNF expression; **d** CCK-8 for cell proliferation; **e**, **e'** Flow cytometry for cell apoptosis; **f**, **f'** Western blot for protein expression of p-TrkB, p-α-syn, PINK1 and DJ-1; **g**–**g'''** Immunofluorescence for the level of α-syn and quantification of the relative fluorescent intensity (scale bar = 20 µm); **h**, **h'** Western blot for protein expression of autophagy-related proteins; **i–i'''** mRFP-GFP-LC3 for autophagic flow and quantification of the relative fluorescent intensity (scale bar = 20 µm). ***p < 0.001, **p < 0.01, *p < 0.05, the same below

We first treated the cells with MPP^+^. CCK-8 assays showed that the cell viability decreased with increasing time and drug concentration (Fig. 1a). After treating SH-SY5Y cells with 1 mM MPP^+^ for 24 h, RT‒qPCR showed that BDNF mRNA levels were reduced in the MPP^+^ group (Fig. 1b). Cells were transfected with BDNF and cultured in medium containing MPP^+^, and the ELISA results showed that MPP^+^ inhibited the secretion of BDNF (Fig. 1c). The CCK-8 and flow cytometry results showed that the cell viability in the MPP^+^ group was substantially reduced, and the cell viability was dramatically enhanced after overexpression of BDNF (Fig. 1d–e'). In addition, the expression levels of phosphorylated TrkB (p-TrkB), PINK1, and DJ-1 proteins decreased, and the expression levels of phosphorylated α-syn (p-α-syn) increased after cells were treated with MPP^+^, which was reversed after overexpression of BDNF (Fig. 1f, f'). The result of α-syn immunofluorescence was consistent with that of Western blotting (Fig. 1g–g'''). The results of autophagy-related proteins showed that the expression of LC3 II/I and Beclin1 was downregulated, the expression of p62 was upregulated, and the autophagy level was decreased in the MPP^+^ group, which was reversed after overexpression of BDNF (Fig. 1h, h'). The autophagic flow activity was decreased in the MPP^+^ group, while autophagic flow activity was elevated after BDNF overexpression (Fig. 1i–i'''). These results suggest that BDNF can promote TrkB phosphorylation and inhibit α-syn Ser129 phosphorylation to inhibit the formation of Lewy bodies. Overexpression of BDNF promotes neuronal cell survival and may be associated with autophagy.

### BDNF alleviates the damage in the PD cell model by mediating autophagy

**Fig. 2:**
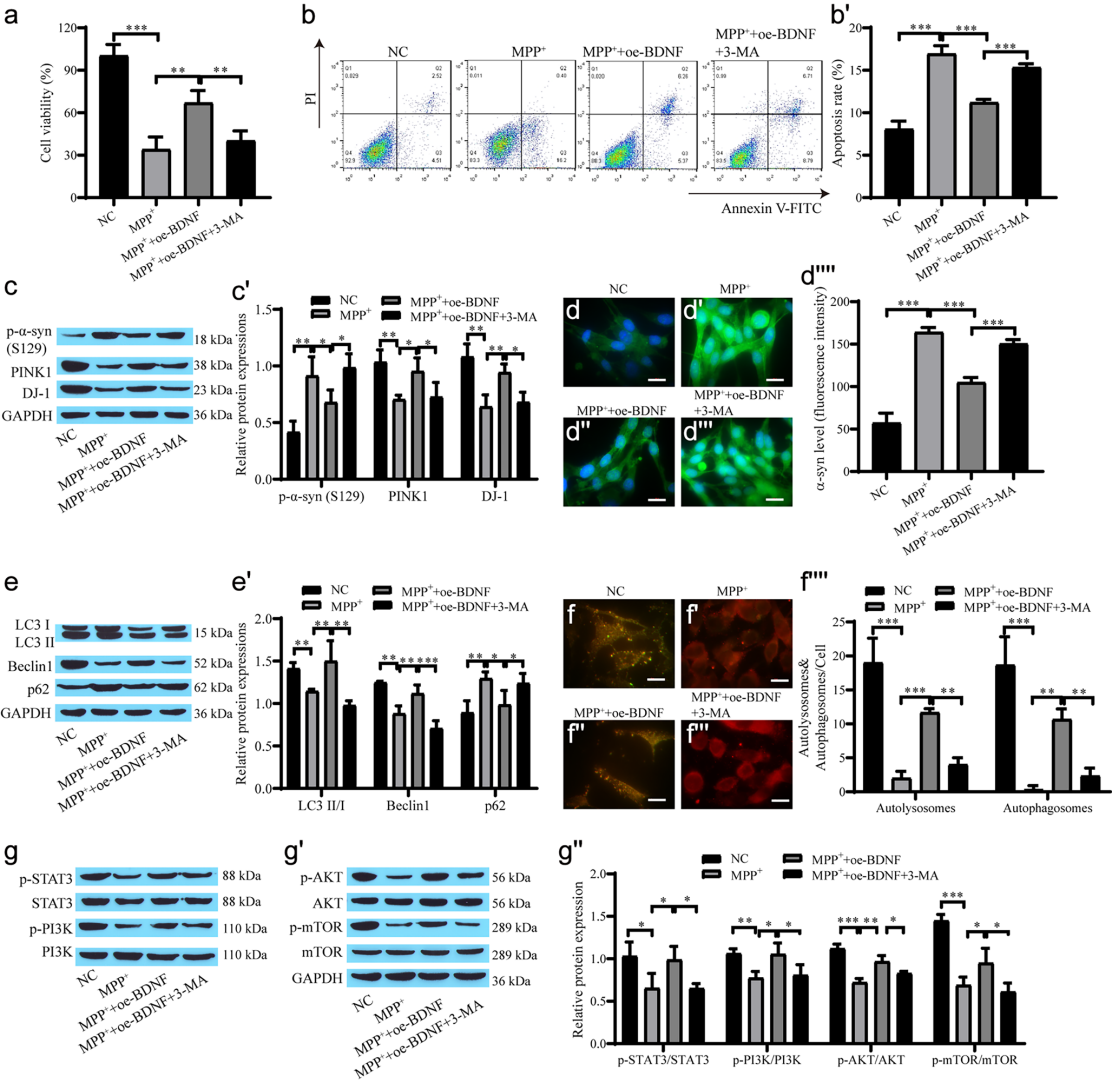
BDNF-mediated autophagy alleviates injury in the PD cell model. **a** CCK-8 for cell proliferation viability; **b**, **b'** Flow cytometry for cell apoptosis; **c**, **c'** Western blot for protein expression of p-α-syn, PINK1 and DJ-1; **d**–**d''''** Immunofluorescence for α-syn and quantification of the relative fluorescent intensity (scale bar = 20 µm); **e**, **e'** Western blot for the level of autophagy-related proteins; **f**–**f''''** mRFP-GFP-LC3 for autophagic flow and quantification of the relative fluorescent intensity (scale bar = 20 µm); **g**–**g''** Western blot for the level of STAT3, p-STAT3, PI3K, p-AKT, p-PI3K, AKT, mTOR and p-mTOR

Next, we verified whether BDNF mediates autophagy to alleviate damage in PD model cells. BDNF was transfected into the cells, and then the cells were cultured with medium containing MPP^+^ and one group was treated with 5 mmol/L 3-MA. CCK-8 and flow cytometry showed that the MPP^+^ group had the lowest proliferation and the highest number of apoptotic cells, while BDNF promoted proliferation and inhibited apoptosis, which showed that BDNF had a protective effect on neuronal cells, while 3-MA inhibited the protective effect of BDNF (Fig. 2a–b'). The Western blot results showed that the expression levels of PINK1 and DJ-1 increased and the expression levels of p-α-syn decreased after overexpression of BDNF, which was reversed after the addition of 3-MA (Fig. 2c, c'). The α-syn immunofluorescence results were consistent with the Western blot results (Fig. 2d–d''''). In addition, compared with the MPP^+^ + oe-BNDF group, the MPP^+^ + oe-BNDF + 3-MA group exhibited substantially decreased protein expression levels of LC3 II/I and Beclin1, and the level of p62 was elevated (Fig. 2e, e'). The mRFP-GFP-LC3 experiment also showed similar results (Fig. 2f–f''''). Western blot analysis showed that the levels of p-STAT3/STAT3, p-AKT/AKT, p-PI3K/PI3K and p-mTOR/mTOR in the MPP^+^ group were lower than those in the NC group, and that BDNF promoted protein phosphorylation. However, 3-MA inhibited the BDNF-promoted protein phosphorylation (Fig. 2g–g''). These results suggest that BDNF has neuroprotective effects through STAT3/PI3K/mTOR-mediated autophagy.

## BDNF promotes STAT3 phosphorylation and STAT3 targets PI3K

**Fig. 3:**
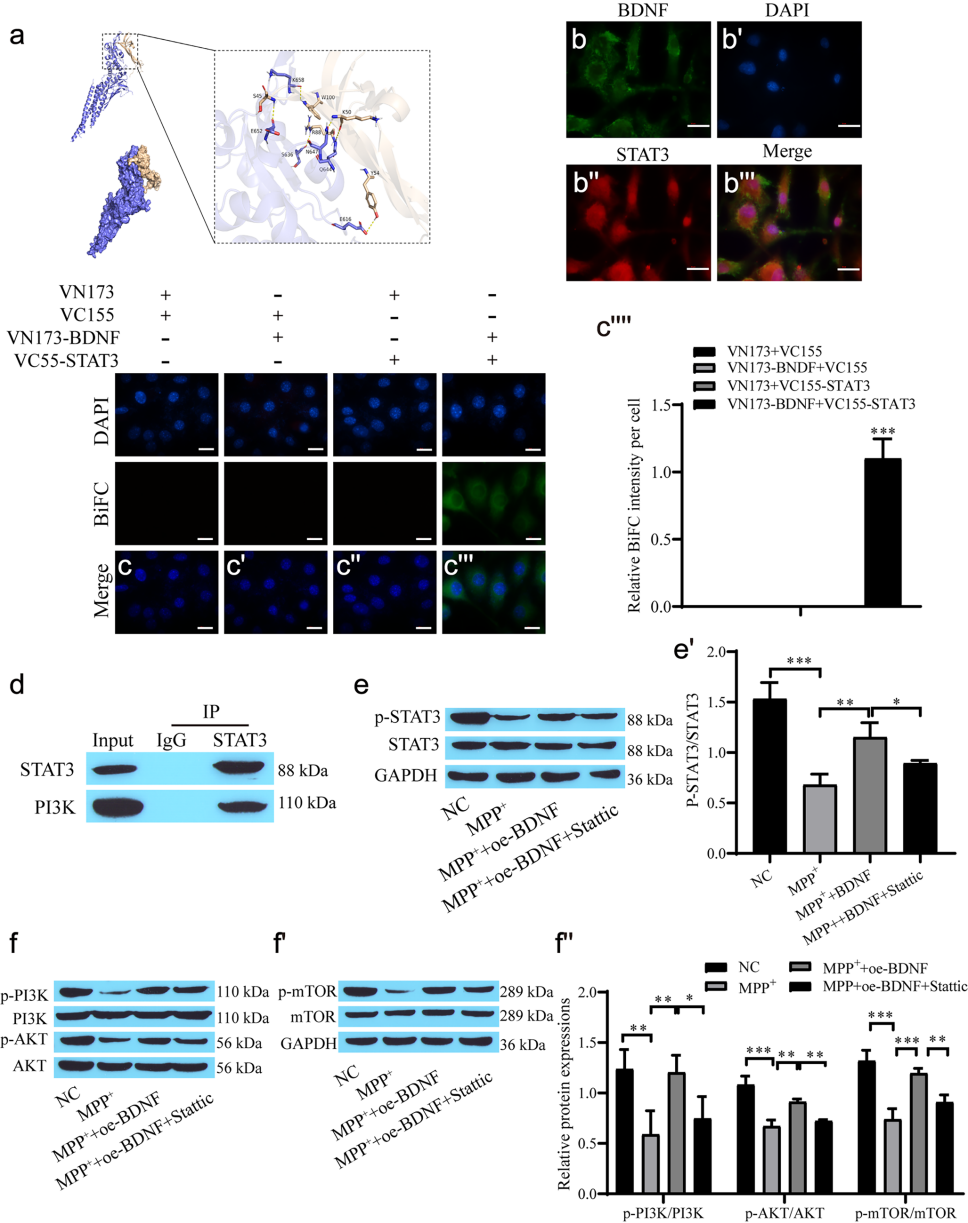
BDNF promotes STAT3 and PI3K phosphorylation. **a** Predicting the binding force of BDNF and STAT3; **b**–**b'''** Immunofluorescence for BDNF and STAT3 expression; **c**–**c''''** BiFC assay for the interaction between BDNF and STAT3 and quantification of the relative fluorescent intensity (scale bar = 20 µm); **d** Co-immunoprecipitation for the interaction between STAT3 and PI3K; **e**–**f''** Western blot showing the levels of STAT3, p-STAT3, PI3K, p-PI3K, AKT, p-AKT, mTOR and p-mTOR

Bioinformatic analysis predicted the binding of BDNF to STAT3 (Fig. 3a). Immunofluorescence results showed that BDNF was expressed in the cytoplasm, while STAT3 was expressed in the nucleus and cytoplasm (Fig. 3b–b'''). Direct physical interaction of BDNF with STAT3 was observed in the BiFC assay (Fig. 3c–c'''''), and the coimmunoprecipitation results indicated an interaction between STAT3 and PI3K (Fig. 3d). Western blot analysis showed that the levels of p-STAT3/STAT3, p-AKT/AKT, p-PI3K/PI3K and p-mTOR/mTOR in the MPP + group were lower than those in the NC group, and BDNF promoted protein phosphorylation. However, Stattic reversed these results (Fig. 3e–f'').

### The PI3K/AKT/mTOR pathway alleviates injury in the PD cell model by mediating autophagy

**Fig. 4:**
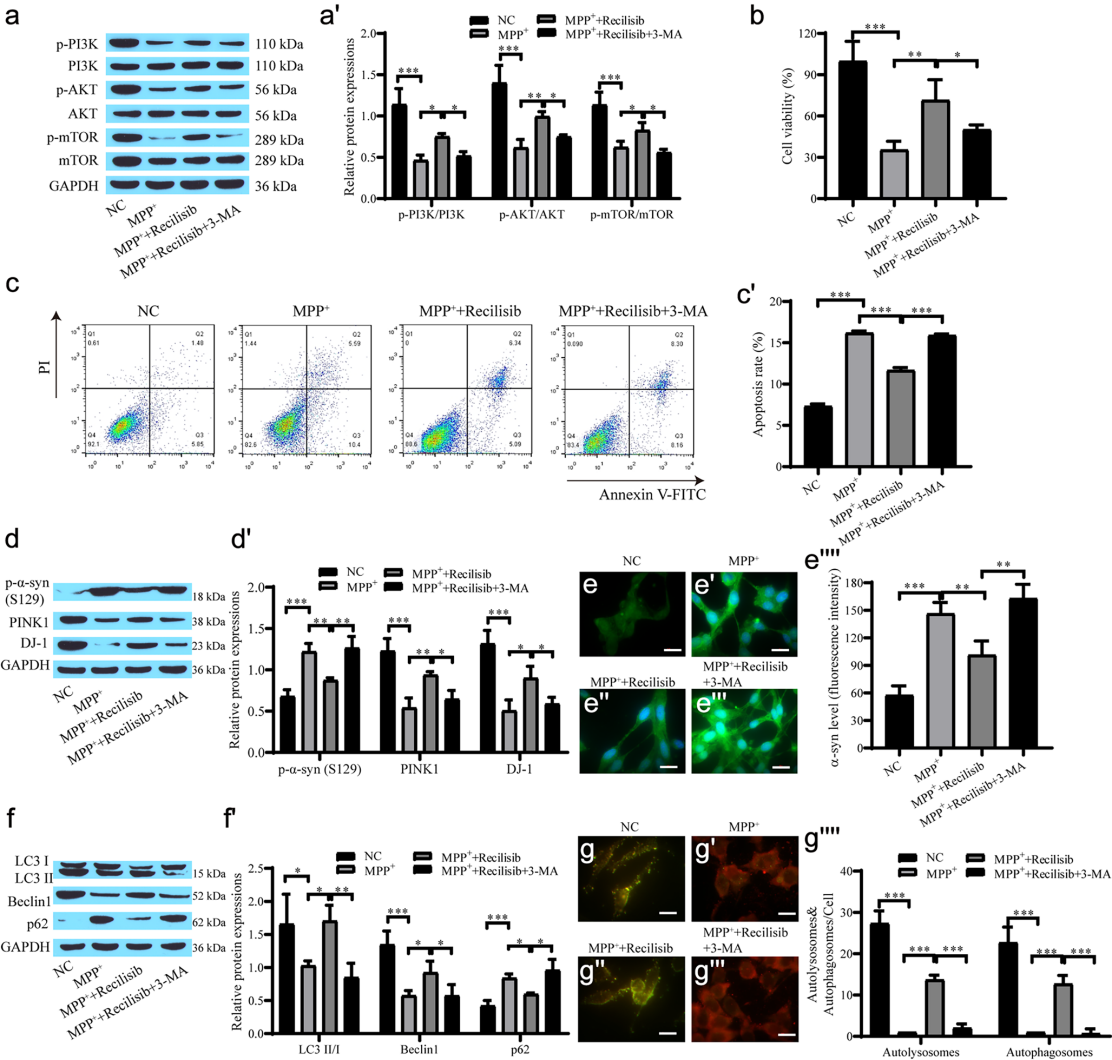
The PI3K/AKT/mTOR pathway alleviates damage in the PD cell model by mediating autophagy. **a**, **a'** Western blot for PI3K, p-PI3K, AKT, p-AKT, mTOR, p-mTOR levels; **b** CCK-8 assay for cell proliferation; **c**, **c'** Flow cytometry for apoptosis; **d**, **d'** Western blot for the level of p-α-syn, PINK1 and DJ-1 proteins; **e**–**e''''** Immunofluorescence for the level of α-syn and quantification of the relative fluorescent intensity (scale bar = 20 µm); **f**, **f'** Western blot for the level of autophagy-related proteins; **g**–**g''''** mRFP-GFP-LC3 for autophagic flow and quantification of the relative fluorescent intensity (scale bar = 20 µm)

We further verified that the PI3K/AKT/mTOR pathway alleviated injury in the PD cell model by mediating autophagy. Recilisib promoted the levels of p-STAT3/STAT3, p-AKT/AKT, p-PI3K/PI3K and p-mTOR/mTOR, which is similar to the overexpression of BDNF. The promotive effect of Recilisib on phosphoprotein expression was inhibited after the addition of 3-MA (Fig. 4a**–**a'). Recilisib promoted cell proliferation, and 3-MA inhibited the effect of Recilisib on cell proliferation (Fig. 4b–c'). Western blot analysis showed that the level of p-α-syn protein in the Recilisib group was observably lower than that in the MPP^+^ group, and the expression levels of PINK1 and DJ-1 increased. 3-MA increased p-α-syn levels, while PINK1 and DJ-1 expression levels decreased (Fig. 4d, d'). The α-syn immunofluorescence results showed that the expression of α-syn was inhibited in the Recilisib group, while the fluorescence intensity of α-syn was increased in the 3-MA group (Fig. 4e–e''''). The Western blot results revealed that the autophagy level was reduced in the MPP^+^ group, while Recilisib promoted autophagy, and 3-MA suppressed autophagy (Fig. 4f–f'). The MRFP-GFP-LC3 results also demonstrated the same pattern (Fig. 4g–g''''). Recilisib promotes autophagy, increases cell viability and reduces cell apoptosis by activating the PI3K/AKT/mTOR pathway, while 3-MA inhibits the protective effect of Recilisib on cells.

### BDNF regulates neuronal autophagy through the STAT3/PI3K/AKT/mTOR signaling axis to alleviate PD model cell injury

**Fig. 5:**
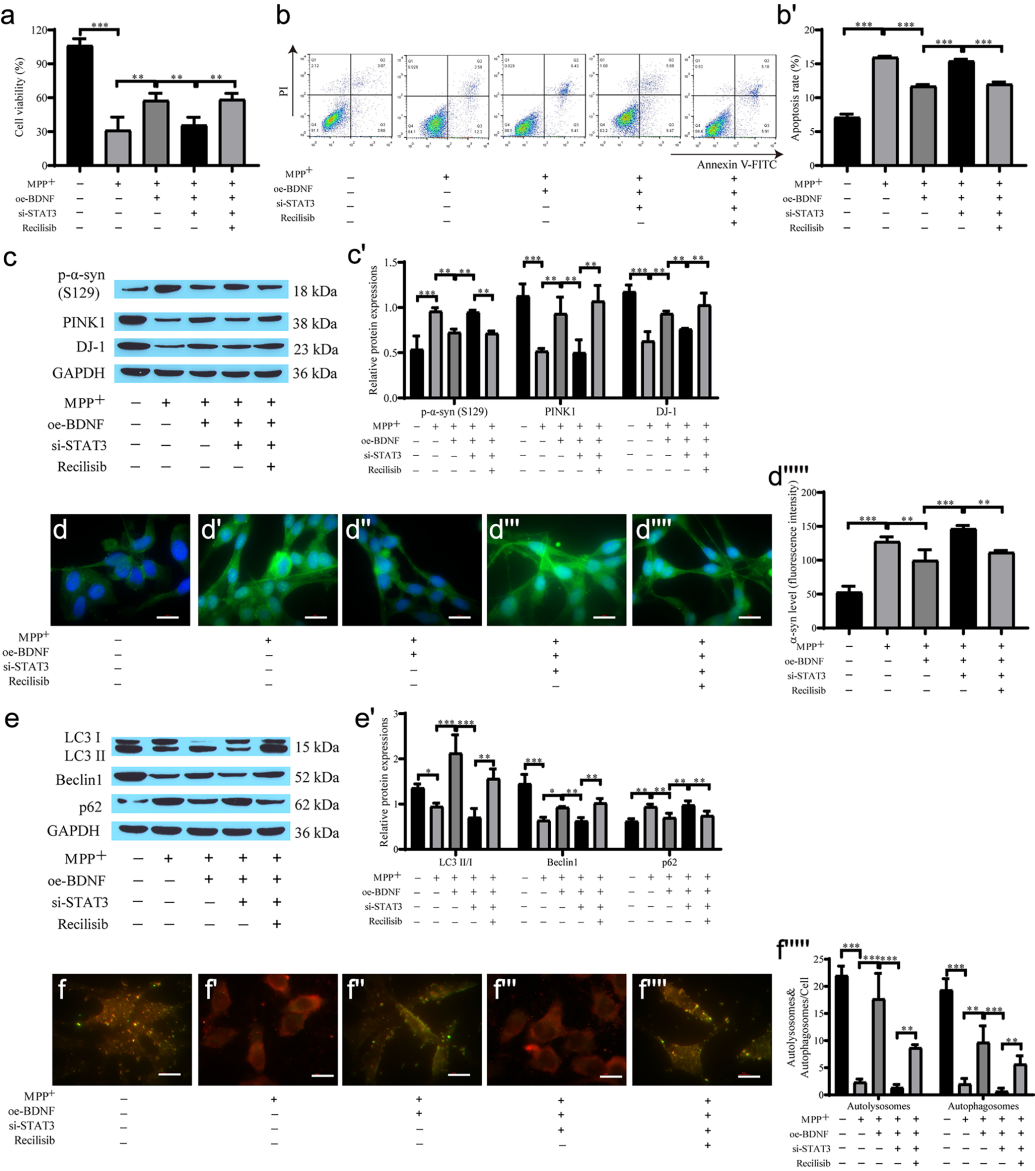
BDNF regulates neuronal autophagy through the STAT3/PI3K/AKT/mTOR signaling axis to alleviate PD model injury. **a** CCK-8 for cell proliferation; **b**, **b'** Flow cytometry for cell apoptosis; **c**, **c'** Western blot for α-syn, PINK1, DJ-1 protein expression; **d**–**d'''''** Immunofluorescence detection of α-syn expression and quantification of the relative fluorescent intensity (scale bar = 20 µm); **e**, **e'** Western blot for the level of autophagy-related protein; **f**–**f'''''** mRFP-GFP-LC3 for autophagic flow and quantification of the relative fluorescent intensity (scale bar = 20 µm)

The CCK-8 and flow cytometry results showed that oe-BDNF restrained MPP^+^-induced cell apoptosis and that si-STAT3 inhibited cell proliferation. Coculture with Recilisib attenuated the inhibitory effect of si-STAT3 on cell proliferation (Fig. 5a–b'). Western blot analysis showed that the level of p-α-syn protein in the oe-BDNF group was dramatically lower than that in the MPP^+^ group, and si-STAT3 weakened the inhibitory effect of oe-BDNF and upregulated p-α-syn expression. However, Recilisib reversed the effect of si-STAT3 on the expression of α-syn, and the expression of PINK1 and DJ-1 was opposite of that of α-syn in each group (Fig. 5c, c'). The immunofluorescence results of α-syn expression showed that the level of α-syn in the oe-BDNF group was reduced compared with that in the MPP^+^ group. The fluorescence intensity in the si-STAT3 group was increased, and the expression of α-syn was also downregulated after the addition of Recilisib (Fig. 5d–d'''''). In addition, autophagy-related protein expression in the MPP^+^ group was downregulated, while oe-BDNF promoted autophagy, and si-STAT3 reversed the promotive effects of BDNF overexpression on autophagy. Recilisib attenuated the inhibitory effect of si-STAT3 on autophagy (Fig. 5e, e'). The mRFP-GFP-LC3 autophagic flow results showed the same pattern (Fig. 5f–f'''''). Therefore, BDNF alleviated neuronal cell injury by promoting autophagy and downregulating the expression of α-syn, while si-STAT3 restrained cell proliferation and promoted apoptosis through the STAT3/PI3K/AKT/mTOR signaling axis.

### BDNF alleviated primary neuron injury through the STAT3/PI3K/AKT/mTOR signaling axis

**Fig. 6:**
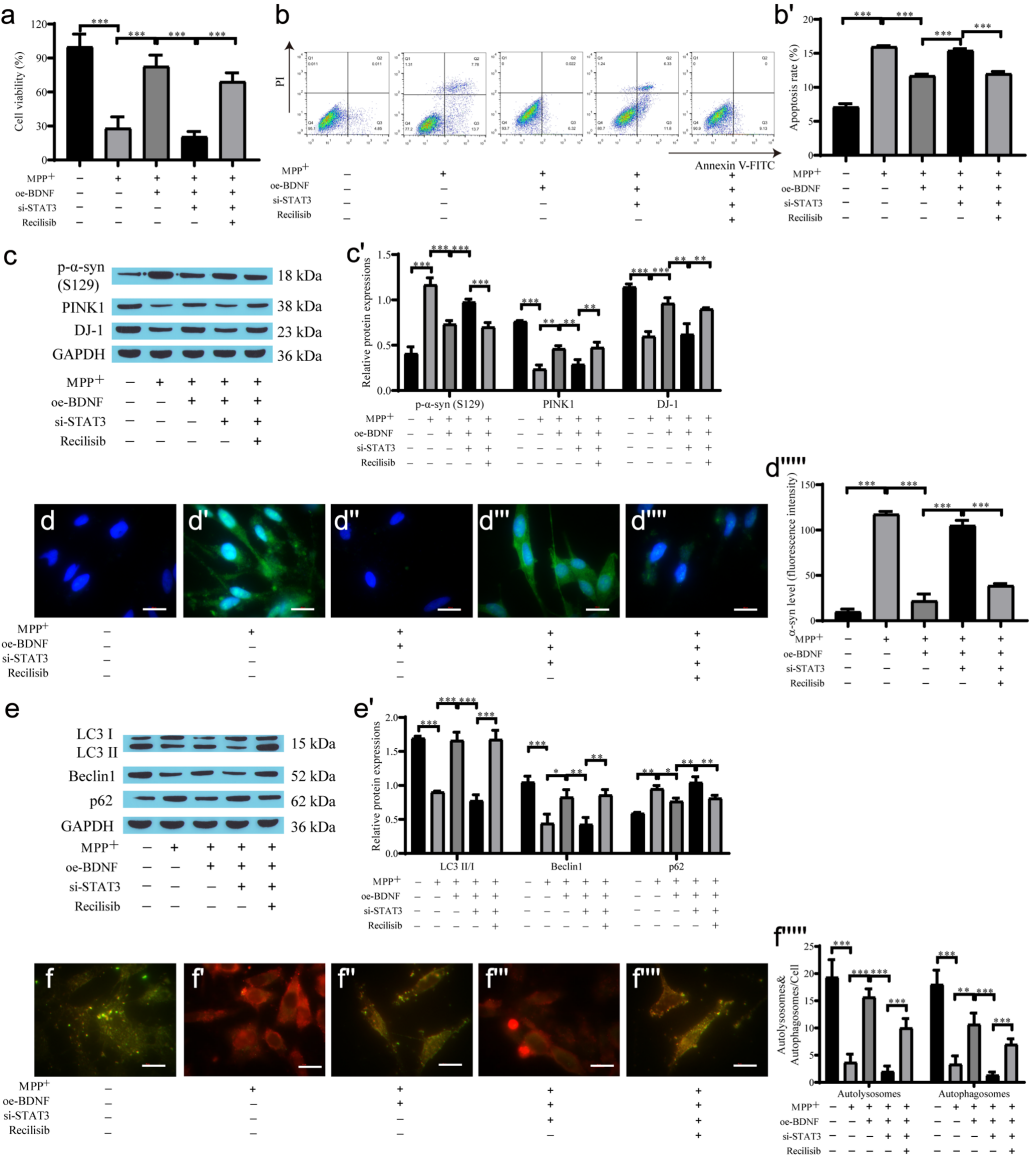
BDNF regulates neuronal autophagy through the STAT3/PI3K/AKT/mTOR signaling axis to alleviate PD model injury. **a** CCK-8 for cell proliferation; **b**, **b'** Flow cytometry for cell apoptosis; **c**, **c'** Western blot for α-syn, PINK1, DJ-1 protein expression; **d**–**d'''''**) Immunofluorescence for α-syn expression and quantification of the relative fluorescent intensity (scale bar = 20 µm); **e**, **e'** Western blot for the level of autophagy-related protein; **f**–**f'''''** mRFP-GFP-LC3 for autophagic flow and quantification of the relative fluorescent intensity (scale bar = 20 µm)

In the aforementioned experiments, we investigated the role and mechanism of BDNF in SH-SY5Y cells. SH-SY5Y cells are widely used to establish PD cell models, but there are still some limitations. Therefore, we obtained primary cultured neurons from embryonic mouse brain tissue for the following in vitro experiments. oe-BDNF reduced MPP^+^-induced cell apoptosis and si-STAT3 inhibited cell proliferation. Coculture with Recilisib attenuated the inhibitory effect of si-STAT3 on cell proliferation (Fig. 6a–b'). Western blot analysis showed that the level of p-α-syn protein in the oe-BDNF group was dramatically lower than that in the MPP^+^ group, and si-STAT3 reversed the inhibitory effect of oe-BDNF and upregulated p-α-syn expression. However, Recilisib reversed the effect of si-STAT3 on the expression of α-syn, and the expression of PINK1 and DJ-1 was opposite of that of α-syn in each group (Fig. 6c, c'). The immunofluorescence results of α-syn expression showed that the level of α-syn in the oe-BDNF group was reduced compared with that in the MPP^+^ group. The fluorescence intensity in the si-STAT3 group was increased, and the expression of α-syn was also downregulated after the addition of Recilisib (Fig. 6d–d'''''). In addition, autophagy-related protein expression in the MPP^+^ group was downregulated, oe-BDNF promoted autophagy, and si-STAT3 reversed the promotive effects of BDNF overexpression on autophagy. Recilisib attenuated the inhibitory effect of si-STAT3 on autophagy (Fig. 6e, e'). The mRFP-GFP-LC3 autophagic flow results showed the same pattern (Fig. 6f−f'''''). Therefore, BDNF similarly alleviated neuronal cell injury by promoting autophagy and downregulating the expression of α-syn, and si-STAT3 reduced cell proliferation and promoted apoptosis through the STAT3/PI3K/AKT/mTOR pathway.

## Animal model verification of the effect of BDNF on alleviating PD pathology

**Fig. 7:**
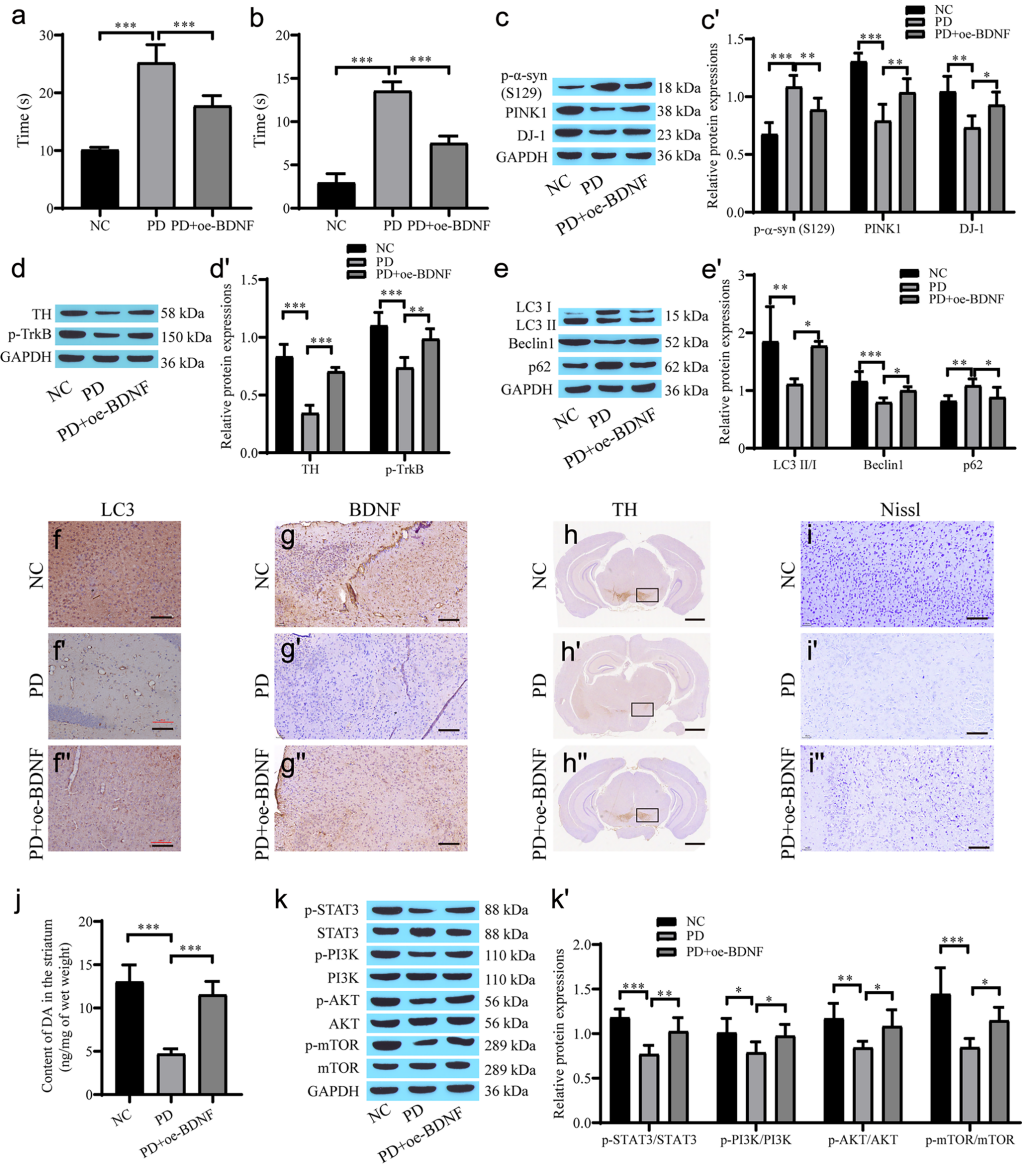
The role of BDNF in alleviating PD pathology was verified using an animal model. **a** A pole climbing experiment for evaluating movement state of the mouse; **b** a grid experiment for evaluating rigidity state of the mouse; **c**, **c'** Western blot for protein expression of p-α-syn, PINK1 and DJ-1 proteins; **d**, **d'** Western blot for the level of TH and p-TrkB proteins; **e**, **e'** Western blot the level of autophagy-related proteins; **f**, **f''** Immunohistochemistry for the level of LC3 (scale bar = 100 µm); **g**–**g''** Immunohistochemistry for the level of BDNF (scale bar = 100 µm); **h**–**h''** Immunohistochemistry for the level of TH (scale bar = 1 mm); **i**–**i''** Nissl stain for neuronal cell survival (scale bar = 100 µm); **j** HPLC for DA content; **k**, **k'** Western blot for protein expression of STAT3, p-STAT3, PI3K, p-PI3K, AKT, p-AKT, mTOR and p-mTOR

MPTP was used to establish a PD mouse model. The movement state and stiffness state of mice were evaluated by the pole climbing test and grid test. The climbing time and stiffness time of the PD group were significantly increased, while the climbing time and stiffness time of the PD + oe-BDNF group were significantly decreased (Fig. 7a, b). The expression of p-α-syn in the PD group was substantially higher than that in the NC group. BDNF reduced the level of p-α-syn, while the levels of p-TrkB, TH1, PINK1 and DJ-1 in the PD group decreased (Fig. 7c–d'). Western blot analysis of autophagy-related proteins revealed that the autophagy level was decreased in the PD group and that oe-BDNF promoted autophagy (Fig. 7e, e'). The immunohistochemistry results showed that TH, BDNF and LC3 were downregulated in the PD-treated mouse brain, and the overexpression of BDNF reversed the expression of these proteins (Fig. 7f–h''). Nissl staining showed that oe-BDNF alleviated MPTP-induced neuronal damage in mice (Fig. 7i–i''). MPTP treatment led to the degeneration and death of dopamine neurons, and BDNF showed modest neuroprotective effects. BDNF rescues MPTP-induced decrease of the DA content in striatum (Fig. 7j). Western blot analysis showed that the levels of p-STAT3, p-PI3K, p-AKT and p-mTOR in the PD group were dramatically lower than those in the NC group, while oe-BDNF upregulated the expression of the phosphorylated proteins (Fig. 7k, k'). Therefore, BDNF can upregulate the expression of STAT3/PI3K/AKT/mTOR phosphorylated proteins, increase the level of autophagy and inhibit neuronal apoptosis, thus alleviating PD pathology.

## Discussion

PD is characterized by the gradual loss of dopaminergic neurons in the SN within the midbrain, which is usually accompanied by the accumulation of Lewy bodies in neuronal cell bodies and Lewy neurites in neuronal processes, and α-synuclein is an important protein component of Lewy bodies (Moudio et al. 2022). There are many risk factors for PD. Moreover, a steady accumulation of damaged proteins and impaired or unconventionally embellished proteins due to proteasome and lysosome dysfunction are common mechanisms for PD (Cacabelos 2017; Hou et al. 2020). There is increasing evidence that mitochondrial homeostasis is closely related to CNS function, and PINK1 (PTEN-induced putative kinase) is one of the key factors in the mitochondrial quality control pathway (Goncalves and Morais 2021). The DJ-1 protein is involved in multiple specific mechanisms that protect dopaminergic neurons against neurodegeneration in PD (Dolgacheva et al. 2019). In the PD cell and animal models we constructed, the expression of α-syn was upregulated, while content of DA, the expression of PINK1 and DJ-1 was downregulated, which supports the above views.

BDNF is an important member of the neurotrophic factor family, which plays a neuronal protective role, is widely expressed in the central nervous system, and participates in neural plasticity and the repair of stress-induced nerve injury (Colucci-D'Amato et al. 2020). An increasing number of studies have attested that BDNF is relevant to the occurrence, development and treatment of neurodegenerative diseases, and it is one of the most studied neurotrophic factors in the field of neurobiology in relation to neurodegenerative diseases (Lima Giacobbo et al. 2019). Studies have shown that BDNF and its downstream pathways play an important role in neurogenesis and synaptic plasticity in the CNS (Keifer 2022). Researchers have been investigating the relationship between BDNF levels and activity and the occurrence and outcome of neurodegenerative diseases. PD Wang et al. (2018) found that BDNF inhibits asparagine endopeptidase activity associated with neurodegenerative diseases through AKT phosphorylation. In addition, Palasz et al. (2020) found that BDNF advances the survival of dopaminergic neurons and increases dopaminergic neurotransmission and kinetic performance in an animal model of PD and that BDNF may be a promising therapeutic agent for the treatment of PD. These findings are consistent with our results.

As one of the dominating degradation pathways in cells, autophagy plays an important role in maintaining the efficient renewal of proteins and organelles in cells (Jimenez-Moreno and Lane 2020). There is an association between impaired autophagy and the accumulation of misfolded or unconventional proteins in PD models, suggesting autophagy as a novel therapeutic target for the treatment of PD. Specifically, upregulation of α-syn is a major pathological hallmark of PD (Spillantini et al. 1997). This upregulation is associated with impaired function of the protein degradation machinery. Autophagy is one of the main ways to degrade α-syn in cells. In this study, it was found that an increase in a-syn levels led to neuronal apoptosis, while an increase in autophagic flux led to a decrease in a-syn protein levels. Overexpression of BDNF promotes TrkB phosphorylation, increases autophagy levels, and inhibits Ser129 phosphorylation of a-syn, thereby inhibiting neuronal apoptosis and alleviating the progression of PD.

STAT3 is located at the intersection of multiple oncogenic signaling pathways, which can facilitate the growth, proliferation, progression and metastasis of tumor cells through multiple channels (Yin et al. 2015). Zhang et al. declared that the level of p-STAT3 was substantially elevated in breast cancer tissues and positively correlated with lymph node metastasis. This study illustrates that phosphorylated STAT3 may be involved in breast cancer cell proliferation and progression (Zhang et al. 2020). The expression of p-STAT3 in non-small-cell lung cance (NSCLC) has also been illustrated to be interrelated with stage, differentiation, lymph node metastasis, and prognosis (Chen et al. 2020). The PI3K/AKT/mTOR signaling pathway plays a key role in regulating cell growth, proliferation, and viability, and mTOR signaling is a central regulator of autophagy (Zhu et al. 2019). In our study, we conducted a BiFC assay and found direct physical interaction of BDNF with STAT3, and the coimmunoprecipitation results indicated an interaction between STAT3 and PI3K. Stattic treatment inhibited STAT3 phosphorylation and suppressed the PI3K/AKT/mTOR pathway.

In this study, we used the pole climbing method and grid experiment to test the behavior of PD model mice. The results showed that compared with those of normal mice, the climbing from the wooden pole to the ground and the time of any paw movement time of PD model mice were significantly increased, indicating that the motor and coordination ability of mice significantly decreased after modeling. After intracerebroventricular injection of the BDNF lentivirus expression vector, the motor and coordination abilities of mice were improved, the number of DA neurons was increased in the PD, and the density of TH-positive fibers was elevated in the striatum. Transfection of BDNF promotes STAT3 phosphorylation to activate the PI3K/AKT/mTOR pathway, promotes autophagy, downregulates α-syn expression, enhances cell proliferation and reduces neuronal apoptosis. These data are consistent with previous research showing that autophagy dysfunction is involved in the pathogenesis of PD via the PI3K/Akt/mTOR signaling pathway (Fakhri et al. 2021; Wang et al. 2020).

## Conclusion

In summary, our study verifies that BDNF affects the process of PD by promoting STAT3 phosphorylation and regulating the level of autophagy in neurons. BDNF has low expression in PD model mice and MPP^+^-treated SH-SY5Y cells. BDNF upregulates the expression of p-STAT3, activates the PI3K/AKT/mTOR pathway, promotes the level of autophagy, reduces the aggregation of α-syn, and relieves the damage to neuronal cells in PD model mice. This study provides an important reference for explaining the potential mechanisms underlying PD pathogenesis and developing new therapeutic targets.

## Data Availability

The datasets used and/or analyzed during the current study are available from the corresponding author upon reasonable request.
